# Role extension in advanced ultrasound practice: A framework approach and case study

**DOI:** 10.1177/1742271X221102577

**Published:** 2022-06-20

**Authors:** Ruth Reeve, Antony Higginson, Christopher Ball, Richard Beable, Mike Smith

**Affiliations:** 1 Portsmouth University Hospital NHS trust, Portsmouth, UK; 2 University of Southampton, Southampton, UK; 3 Cardiff University, Cardiff, UK

**Keywords:** Ultrasound education, audit, role development, sonographers, competency, safety, governance, framework

## Abstract

**Introduction:**

Role extension into novel areas of ultrasound practice can be challenging for health care professionals. Expansion into existing areas of advanced practice typically occurs using established processes and accredited training; however, in areas where there is no formal training, there can be a lack of support for how to develop new and progressive clinical roles.

**Topic Description:**

This article presents how the use of a framework approach for establishing areas of advanced practice can support individuals and departments with safely and successfully developing new roles in ultrasound. The authors illustrate this via the example of a gastrointestinal ultrasound role, developed in an NHS department.

**Discussion:**

The framework approach comprises three elements, each interdependent upon and inform each other: (A) Scope of practice, (B) Education and competency and (C) Governance. (A) Defines (and communicates) the role extension and area(s) of subsequent ultrasound imaging, interpretation and reporting. By identifying the why, how and what is required this informs (B) the education and assessment of competency for those taking on new roles or areas of expertise. (C) Is informed by (A) and is an ongoing process of quality assurance to safeguard high standards in clinical care. In supporting role extension, this approach can facilitate new workforce configurations, skill expansion and enable increasing service demands to be met.

**Summary:**

By defining and aligning the components of scope of practice, education/competency and governance, role development in ultrasound can be initiated and sustained. Role extension utilising this approach brings benefits for patients, clinicians and departments.

## Introduction

The technological advancements and recognised benefits of ultrasound have resulted in an increasing demand for ultrasound services worldwide.^1^ With an ongoing shortage of radiologists, health care professionals have been developing and extending their ultrasound skills in order to enhance the ultrasound workforce and meet service demand.^2,3^ In health care organisations within the United Kingdom and Europe, allied health professionals and non-radiological specialist doctors have been increasingly developing expertise in ultrasound.^2–4^ The title sonographer is often used to refer to such individuals who undertake and report on ultrasound examinations.

The expansion of practitioners into advanced ultrasound practice has been met with resistance in some countries.^2–5^ Barriers to ‘sonographers’ include resistance from radiologists, inadequate training in ultrasound and a lack of legal framework.^6^ In the United Kingdom, the development of allied health professionals, such as radiographers, into advanced ultrasound practice has become an opportunity for career development. This has been more commonly seen where the development of accredited training and qualifications has resulted in better governance and acceptance of role development.^6^ Outside of the United Kingdom, ultrasound practice can vary widely; for example, in many parts of Europe and Australasia, sonographers may be expected to carry out ultrasound examinations and provide preliminary reports but with radiologists overseeing the report and even writing this independently from the sonographers’ impression.^5^ In the United Kingdom, the National Health Service (NHS) recognises the opportunity of developing advanced sonographer skills and has promoted a four-tier approach to advance their careers through role extension.^7^ Thus, leading the way in creating and providing pathways for sonographers to take on increasingly advanced roles to meet the developing clinical, educational and research demands of services.^2,3^

Across health care organisations such as the NHS, many ultrasound practitioners/sonographers begin their careers working in general abdominal, gynaecology and obstetric ultrasound. However, there are areas of more established role extension for ultrasound practitioners, seen in musculoskeletal, head and neck and breast ultrasound, with specific accredited postgraduate training and qualifications.^6,8,9^ Despite this role extension in certain areas of ultrasound, there still remains challenges in other specialist areas that are less well recognised, or in developing areas of ultrasound such as gastrointestinal (GI) ultrasound. In areas with no formal training pathway, this has become a challenge for those who wish to develop ultrasound skills into such specialist areas.

This article discusses the opportunity of role progression in ultrasound, outlining a framework used for developing extended roles, and the use of an accompanying case study highlighting how individuals may use the framework approach to consolidate and expand areas of advanced practice and new areas of imaging.

## Framework approach

**Figure 1.**
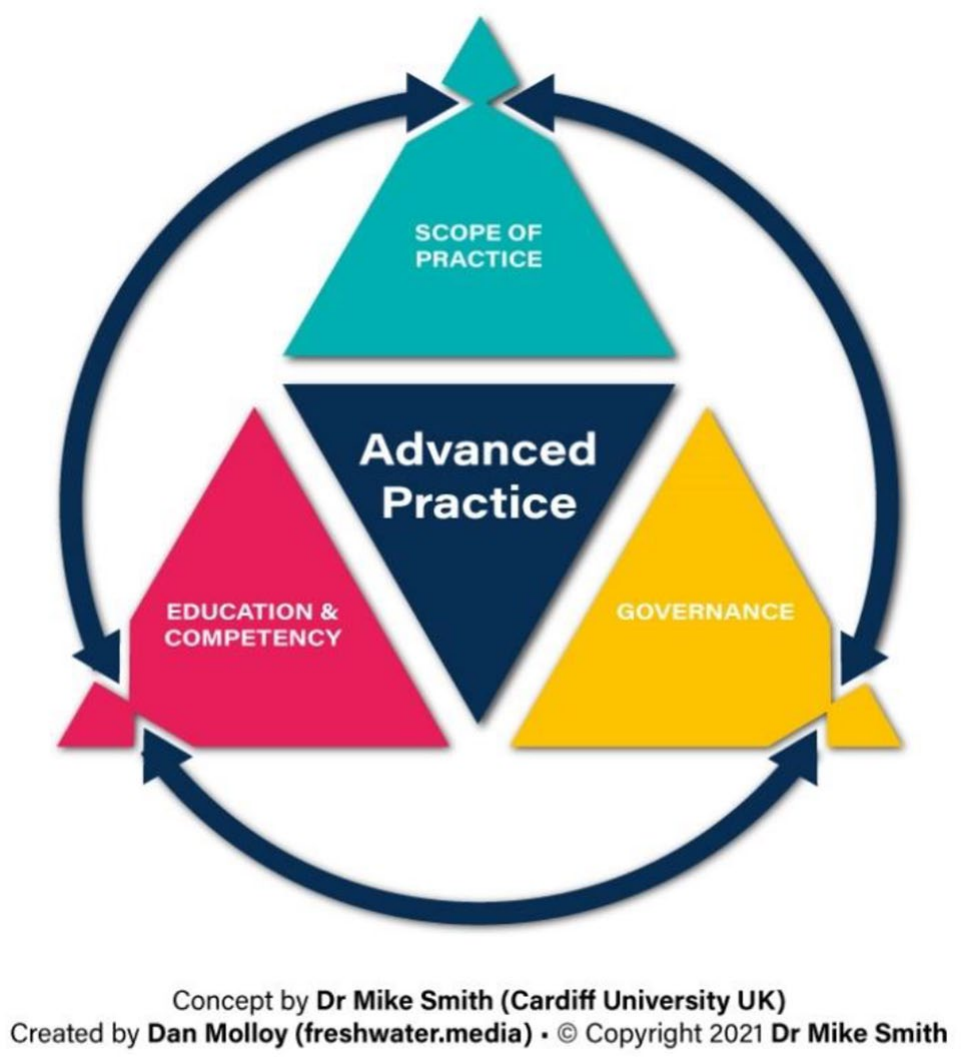
Framework approach to consolidating or expanding sonographer roles.

For areas of novel role development, a framework approach can be used to provide a robust foundation for the development and consolidation of such practice. The framework approach comprises the elements of (a) scope of practice (ScoP), (b) education and competency and (c) governance. These terms are well established in the published literature, having been described by authors such as Ambasta et al.,^10^ LoPresti et al.,^11^ Lee and DeCara^12^ and Teunissen et al.^13^ The framework concept is that each of the elements inform and must be in alignment with each other.^14^ It was originally developed by one of the authors to support point of care ultrasound (PoCUS) across areas as diverse as lung ultrasound^14^ and pelvic floor ultrasound.^15^ This has been adapted to support novel role development in sonography. Figure 1 provides a visual representation of the framework approach, with definition and explanation of the terms summarised in Table 1.

**Table 1.** Explanation of scope of practice, education/competency and governance.

Term	Key elements	Additional information
Scope of practice (ScoP)	Refers to the context and scope of the ultrasound imaging performed, *plus* the interpretation/reporting of that ultrasound imaging, *plus* the clinical decision making informed by that ultrasound imaging.	ScoP allows for specifying any ultrasound imaging that is *not* going to be performed; and/or where ultrasound imaging is performed any interpretation/reporting *not* undertaken; and/or where ultrasound imaging is performed any clinical decision making *not* informed by the ultrasound imaging.
Education and competency	Refers to the education undertaken (both informally and formally) and subsequent assessment of competency.	Transparent, purposeful and efficient education provision and competency assessments are made possible by aligning with the ScoP. Appropriate education and competency are key contributors to safety and governance.
Governance	Refers to the processes and practices that allow accountability for continuous improvement of quality of services and safeguarding standards of care.	These are in part informed by the ScoP and by local agreements. Provides a precedent for wider uptake. Includes agreement of professionals who originally provide this imaging, and of other members of the care pathway. Also includes insurance arrangements and quality assurance mechanisms.

### Scope of practice

This component of the framework involves identifying and defining the clinical and sonographic scope of practice that the professional (or profession) will perform. For a new area of practice, this will involve discussion with and agreement of stakeholders as to what the role does and does not involve. This enables identification of the training requirements needed by that professional (or profession) – as the foundation for demonstrating competency in this new role.

### Education and competency

The very nature of advanced practice means that there may not yet be formal training routes available, and this requires a creative and multifaceted approach to training and competency. One strategy is to identify a pre-existing course, which includes a formal competency assessment, and to map the ScoP to it. Such a course may be intended for other imaging professionals; or professionals who specialise in that clinical area (e.g. a Cardiologist, point of care/PoCUS user). Where such an approach is not possible or viable, in-house training may be deemed appropriate. One caveat here is that the professional would require a high level of pre-existing sonographic knowledge and expertise, for example, Postgraduate Diploma or Masters level – upon which the ‘advanced practice-specific’ learning can be overlaid. Competency assessment could take various forms, including audit against imaging findings of an established expert, histopathology results and multidisciplinary team (MDT) outcomes – depending on the relevance to the specific area of advanced practice. Regardless of the route(s) taken, availability of a suitably experienced mentor is essential for direct observation of technique performance (and adaptation), differential sonographic diagnosis and so on.

### Governance

In the United Kingdom, ultrasound imaging as a modality is unregulated and there is no protection of title for the profession of sonography.^6,16^ Appropriate governance is therefore essential for an extended role in ultrasound, in order to support the individual and ensure the provision of safe services for patients.

One aspect of governance in new role development is establishing and formalising an individual’s permissions (professional and management/other members of care pathway) and insurance considerations, to protect the health care professional and patients. From our personal observations, this generally occurs on a case-by-case basis when setting up a service or role development, thus requiring careful consideration. Support from organisations, such as the British Medical Ultrasound Society and the Society and College of Radiographers,^17^ has been growing in recent years to support role development and provide industrial advice. Those who are interested in role expansion are advised to seek advice with such organisations for professional support.

Role expansion often leads to working in areas that during initial training are outside of existing scope of practices and job descriptions. For insurance and professional liability purposes, discussions surrounding indemnity are advised. This is to protect individuals within this role and as such it is important that new roles and responsibilities are reflected within job descriptions and scope of practice documentations. This article does not detail how this may be developed; however, examples from the case study have been highlighted in Table 3. It is critical to consider the importance of adequate governance, where permissions should be clear within the ScoP and reflected in the job title and description.

**Table 2.** GI Sonographer scope of practice.

Indicative imaging performed	Role of the imaging of these structures
^∆^ = Recognition of normal gastrointestinal structures and adjacent organs as landmarks • Liver, pancreas, gallbladder, biliary tracts • Colon, appendix, ileocolic junction, ileum, jejunum, duodenum and stomach	Awareness of spectrum of ‘normal’ presentations Landmark identification serves as mechanism to enhance accuracy of imaging; integral aspect of protocol-based imaging
^◊^ = Identification of ultrasound appearances of normal colon and small bowel including: • Sonographic differences in the varying aspects of the GI tract • Peristalsis and wall spasm • Normal variation of bowel position, including long mesenteric siting of the small bowel, caecum, transverse and sigmoid colon	Awareness of ‘normal’ presentations
Recognition of lesions involving the GI tract including malignant processes: • Appearances of bowel wall thickening • Appearances suggesting malignant transformation • Demonstration of GI tract lesions such as clinically significant polyps and lesions	Building upon ^∆^ and ^◊^: sonographic differential diagnosis, description and (where appropriate) estimation of malignant features
Recognition of GI tract inflammation: • Identify and report ultrasound appearances of appendicitis • Identify and report ultrasound appearances of colonic inflammation including colitis and diverticulitis • Identify and report ultrasound appearances of small bowel inflammation, including ileitis and Crohn’s disease • Identify and report ultrasound appearances of inflammation that may change the normal appearances of the GI tract, such as mesenteric panniculitis and epiploic appendagitis	Building upon ^∆^ and ^◊^: sonographic differential diagnosis and description of GI tract inflammation
Recognition of hepatopancreatobiliary diseases: • Identify and report advanced ultrasound appearances of the liver including diffuse and focal liver pathology, undertake elastography and other advanced techniques to assess liver disease • Identify and report advanced ultrasound appearances of the pancreas including pancreatic cystic lesions under surveillance for malignant potential • Identify and report advanced ultrasound appearances of the biliary tract, including diagnosis of choledocholithiasis	Building upon ^∆^: sonographic differential diagnosis and description of hepatopancreatobiliary disease

GI: gastrointestinal.

**Table 3.** Case study framework for clinical specialist GI sonographer.

Framework item		
Scope of practice	Education and competency	Governance
Bowel ultrasound technique is not included routinely in postgraduate courses and therefore is generally learnt only by qualified sonographers seeking to specialise in this area. Nonetheless, many do possess the core skills required for identifying appendicitis on acute and/or in-patient lists. The expectations of those working under the extended role are to perform, evaluate and report ultrasound examinations at the level of consultant GI radiologists with expertise in bowel ultrasound. Full details of what areas of practice this includes is seen within Table 2.	Based on local experience, a minimum number of hands-on ultrasound sessions (suggested 50 sessions or 200 scans) was undertaken with a specialist. During these sessions, the trainee was expected to participate in the practical aspects of ultrasound scanning, cross-sectional image interpretation and reporting with supervision by a GI ultrasound specialist. In-house clinical education and training was delivered through one-on-one and group training with radiology trainees. In addition, self-directed learning through online resources (such as e-learning for health modules) and participating in research activity helped to develop a critical and advanced knowledge of ultrasound and image interpretation. A record of continued professional development (CPD) activity included bowel ultrasound study days and conference attendance with GI tract education. Attendance at relevant multidisciplinary team meetings with a view to leading on appropriate meetings (Hepatopancreatobiliary, Colorectal and Inflammatory bowel disease). Passed an aptitude skills and report assessment with specialist GI Consultant. Conducted self-directed outcome-related audits assessing accuracy of report findings, compared to histopathology and MDT outcomes.	Encompassing seven pillars of clinical governance, several processes have been put into place. Signed scope of practice document to trust clinical directors’ board level, ensuring the individual is supported by the leadership and management team to work beyond their normal practice locally. To ensure appropriate risk management, details of the individual’s role, insurance arrangements and a description of methods to ensure maintenance of competency. Patient experience is monitored through frequent feedback and reviewal of any complaints. Clinical effectiveness is monitored through continuous monthly personal and peer audit of reporting accuracy undertaken to include a minimum of 10% of reports. Further audit of report accuracy against clinical and pathology findings conducted periodically. These audits ensure clinical effectiveness standards are met and maintained, and that excellent care is being delivered. Any areas highlighted for improvement through audit or clinical feedback are addressed, where systems are in place for continuous improvement (education and training).

GI: gastrointestinal; MDT: multidisciplinary team.

ScoP and competency are essential for not only developing new roles within ultrasound but also for ongoing governance in order to ensure accountability for those within the roles and to ensure continuous improvement for the quality of the service.^18^ Clinical governance is upheld by seven key pillars^19^ which are clinical audit, clinical effectiveness, risk management, openness in the use of clinical information, education and training, staff management, and the patient experience. We suggest that during development and implementation of new roles in ultrasound practice, a phased implementation is applied. This could be using continuous audit cycles to provide a clear process that manages risk, provides openness, identifies areas for further education/training and enables those undertaking the audit to develop and retain confidence with their skills. This in turn can help to build confidence for referring clinicians.

#### Case study: role extension into GI imaging using the framework approach

Until recently, trans-abdominal ultrasound was rarely used for bowel assessment due to challenges visualising the target tissues at sufficient resolution. Instead, endoscopy, magnetic resonance imaging (MRI), computed tomography (CT) and conventional radiography have been the preferred imaging methods.^20^ However, over the past few years, technological advancements and the increasing experience of ultrasound practitioners have meant that ultrasound is now an important tool for visualising bowel pathology. This gives practitioners the ability to diagnose a range of different pathologies such as colorectal tumours and bowel inflammation.^20,21^

In a large district general hospital within a university NHS trust, bowel ultrasound services were developed as a first line and surveillance test for suspected bowel inflammation such as Crohn’s disease. Notable advantages included it being a sensitive, safe and inexpensive diagnostic tool. However, the demand for this service was greater than the capacity available for the specialist GI Radiologists. Training was therefore developed with support from clinical managers, the Radiologists and clinicians to establish a new role in performing GI ultrasound.

The example below details the first role extension of a GI sonographer identified within the published literature and how this role was developed. Following initial development, trial and successful role implementation, the Trust has extended the programme to train a further GI sonographer.

#### Scope of practice

A scope of practice was defined by assessing the demands and clinical needs of the department. Through an iterative process, multiple scope of practice elements were developed by targeting the specific areas of additional ultrasound practice and reporting required (Table 2). The aspects of the scope of practice were based upon the existing practice of GI radiologists and standards set by the Royal College of Radiologists.

As per Table 1, scope of practice not only defines what is included within the individual’s role but also what is not included. For the GI ultrasound role, an ability to scan solid upper abdominal viscera is required as a baseline prior to role expansion. However, during GI ultrasound examinations, the individual performing the scan is undertaking a focussed examination. As such, the scan cannot be relied upon to confirm or exclude elements outside of the expertise of a GI sonographer (e.g. Musculoskeletal (MSK) and vascular anomalies) or tissues outside of the target regions (e.g. gynaecological structures during small bowel assessment). Communication to referring clinicians and the patient of the scope of the scan means that such limitations are transparent.

#### Education and competency

Due to the low usage of GI ultrasound, there was no formal training route available. To develop the necessary skills, a mixture of in-house training and education to develop the required practical and clinical skills was developed. In addition, funding to attend day courses and conferences on GI ultrasound was sourced.

Assessment of skills was undertaken by highly experienced ultrasound practitioners (GI radiologists), with competency being obtained following significant hands-on training and assessment as detailed in Table 3.

Where possible, correlation and outcome-related audit against clinical findings and histopathology was undertaken post hoc to assess the accuracy of the GI ultrasound reporting. Outcome-related audits can be useful in demonstrating the accuracy of ultrasound findings to identify areas for further education, training or research.^16^ For GI ultrasound, it was useful in several clinical areas, such as identifying ultrasound report accuracy of appendicitis. Outcome-related audits for GI specialist sonographers were therefore undertaken using a clear objective, for example, ‘what is the diagnostic accuracy of acute appendicitis?’. The use of a comparator (histopathology and surgical notes), well-defined acceptability level (95% positive predictive value) and assessment criteria (ultrasound report compared to histopathology) helped to identify the competency and accuracy of individuals developing new clinical expertise. Where acceptability was not at the set standard, further training and development could then be targeted to improve the individual’s skills.

#### Governance

Ongoing review of images, report accuracy and CPD is the responsibility of the individual, supervising clinician and department to ensure safe working practices are being achieved. For the GI ultrasound sonographer, this involved continuous audit of findings against pathology, follow-up of cases with clinicians and reported patient outcomes.

#### Benefits to role extension and the framework approach

The benefit of the framework approach is that stakeholders involved in developing the role can identify the desired outcomes and benefits from the role conception. Therefore, measuring outcomes and benefits from the role extension is easy and reproducible (e.g. reducing waiting lists and/or improving local diagnostic accuracy). From our experiences, there are also several non-clinical benefits from role development encompassing leadership, education and research.

Shortages of radiologists create not only opportunities for individuals to extend their roles^2,3^ but also a challenge for the training of ultrasound practitioners. In areas where there is no formal training for expert ultrasound skills, more creative methods may be required to get the experience of working with experienced experts in ultrasound practice. The benefits of training non-radiologists into extended ultrasound roles, however, bring the possibilities for not only easing the burden of the radiology but also by providing MDT training for the next generation of ultrasound experts.

Development of roles into advancing clinical areas requires individuals to extend their knowledge beyond their current scope of practice and previous training and competency, often through working with the wider MDT to gain the required clinical understanding. Involvement in MDT meetings and clinical specialist knowledge brings opportunities for leadership, such as participating in and leading MDT meetings, providing expert opinions and insights to the team to improve care to patient populations. MDT working is identified within the framework approach where the additional benefits include increasing the working relationships for the individual as well as the service itself, bringing opportunities for collaborative projects and research.

The educational aspect of the development framework creates opportunities to identify areas for further evidence base and research. Involvement in research in a clinical environment can lead to clinical academic careers for expert ultrasound practitioners. The benefits of clinical academics include changing and improving care led by research and evidence-based practice.^22^ Combining clinical expertise and research roles allows a transfer of knowledge, innovation and practices across a ‘theory-practice gap’, resulting in several rewards for patients, NHS organisations and individuals.^22^

Those taking on extended roles into new clinical areas may be joining or becoming pioneers in their clinical field, of which comes with expectations to educate others and become leaders in their area of clinical practice. The benefit of utilising the cyclical framework of role development is that it gives individuals the understanding of the requirements needed to gain competency, thus giving insight in how to educate others and be a leading advocate for the specialist area of practice.

#### Challenges to role extension through framework approach

Developing innovative roles in ultrasound practice can be difficult and met with resistance from both clinicians and/or managers who may be unable to visualise or understand the benefits to service users and patient pathways. However, support for developing new roles is essential in planning and training of individuals, both from clinical and managerial supervisors. As demonstrated within the literature, there remain barriers for ultrasound practitioners such as resistance from the existing radiology workforce.^6^ Protectionism and lack of respect have been issues radiographers, in particular, have been facing when developing into various areas of advance practice in ultrasound.^5^ Individuals within an extended role are therefore required to earn respect from their peers and other clinicians, demonstrating reliability and accuracy over time. As a result, mutual respect between all parties involved is essential in maintaining effective and supportive environments to work in and ensure individuals involved remain motivated in developing knowledge and responsibility.^5^

The ongoing national sonographer workforce deficit and increasing demand for ultrasound services presents a challenge for individuals developing new extended roles.^1^ As we have identified, successful role development involves education and clinical governance outside of service delivery, where securing protected time in developing roles to carry out the educational and governance items both during training and in established roles in the face of workforce and service demands presents an important challenge. To develop new skills required for role extension and ensure ongoing governance and education, adequate and detailed job planning that involves non-clinical duties is essential for safe working and should not be underestimated when organising the sustainable development of extended roles within ultrasound. From the experiences developing a GI specialist ultrasound role, support from management, clinical supervisors (e.g. radiologists) and clinicians to support responsibilities outside of direct service delivery is essential to create well-rounded specialist practitioners that meet the pillars of advanced and/or consultant practice.

## Conclusion

Role development in ultrasound is becoming an increasingly frequent necessity and requirement to meet the growing demand for ultrasound services, which is well received and desired by ultrasound practitioners. In areas where there are no established educational or assessment criteria for role extension, we propose that using audit methodology and a framework approach as described in this article individuals and departments can develop reproducible successful structures for developing roles for ultrasound practitioners.

## References

[1] Mitchell P Nightingale J . Sonography culture: power and protectionism. Radiography 2019; 25: 227–234. 31301780 10.1016/j.radi.2019.02.004

[2] Harrison G Martins dos Santos R Kraus B , et al. Radiographers in ultrasound: motivation and role expansion. A survey of European Federation of Radiographer Societies (EFRS). Radiography 2021; 27: 1185–1191. 34294506 10.1016/j.radi.2021.07.003

[3] European Society of Radiology (ESR). International Summit 2014: organisation of clinical ultrasound in the world. Insights Imag 2014; 5: 641–644. 10.1007/s13244-014-0358-9 PMC4263807 25373877

[4] Dietrich CF Sirlin CB O’Boyle M , et al. Editorial on the current role of ultrasound. Appl Sci 2019; 9: 3512.

[5] Harrison G Kraus B Martins Dos Santos R , et al. The role of radiographers in ultrasound: a survey of the national societies within the European Federation of Radiographer Societies (EFRS). Radiography 2021; 27: 761–767. 33658166 10.1016/j.radi.2021.02.003

[6] Hudson D . Reflections on leadership in advanced and consultant radiographic practice within the UK. J Med Imag Radiat Sci 2021; 52: 164–171. 10.1016/j.jmir.2021.02.003 33648876

[7] Dalili D Carne A MacKay J , et al. Musculoskeletal ultrasound imaging standards in the UK: British Society of Skeletal Radiologists (BSSR) position statement. British J Radiol 2021; 94: 20210198. 10.1259/bjr.20210198 PMC8506188 33793317

[8] Sonography. Health Education England, https://www.hee.nhs.uk/our-work/sonography (accessed 24 February 2022).

[9] Halligan A . Implementing clinical governance: turning vision into reality. BMJ 2001; 322: 1413–1417. 11397753 10.1136/bmj.322.7299.1413 PMC1120478

[10] Ambasta A Balan M Mayette M , et al. Education indicators for internal medicine point-of-care ultrasound: a consensus report from the Canadian Internal Medicine Ultrasound (CIMUS) group. J Gen Intern Med 2019; 34: 2123–2129. 31240603 10.1007/s11606-019-05124-1 PMC6816798

[11] LoPresti CM Schnobrich DJ Dversdal RK , et al. A road map for point-of-care ultrasound training in internal medicine residency. Ultrasound J 2019; 11: 10. 31359161 10.1186/s13089-019-0124-9 PMC6638610

[12] Lee L DeCara JM . Point-of-care ultrasound. Curr Cardiol Rep 2020; 22: 149. 32944835 10.1007/s11886-020-01394-y PMC7498117

[13] Teunissen PW Watling CJ Schrewe B , et al. Contextual competence: how residents develop competent performance in new settings. Med Edu 2021; 55: 1100–1109. 10.1111/medu.14517 PMC8451833 33630305

[14] Smith M Hayward S Innes S . A proposed framework for point of care lung ultrasound by respiratory physiotherapists: scope of practice, education and governance. Ultrasound J. 2022. DOI: 10.1186/s13089-022-00266-6. PMC9201799 35708815

[15] Smith M Donnelly G Berry L , et al. Point of care ultrasound in pelvic health: scope of practice, education and governance for physiotherapists. Intern Urogynecol J. Epub ahead of print 12 May 2022. DOI: 10.1007/s00192-022-05200-x. PMC9477927 35552775

[16] Miles N Cowling C Lawson C . The role of the sonographer: an investigation into the scope of practice for the sonographer internationally. Radiography 2022; 28: 39–47. 34391655 10.1016/j.radi.2021.07.017

[17] SoR BMUS Guidelines for Professional Ultrasound Practice SoR, 2021, https://www.sor.org/learning-advice/professional-body-guidance-and-publications/documents-and-publications/policy-guidance-document-library/sor-and-bmus-guidelines-for-professional-ultrasoun (accessed 24 February 2022).

[18] Gray C . What is clinical governance? BMJ 2005; 330: s254.

[19] Maconi G Nylund K Ripolles T , et al. EFSUMB recommendations and clinical guidelines for intestinal ultrasound (GIUS) in inflammatory bowel diseases. Europ J Ultrasound 2018; 39: 304–317. 10.1055/s-0043-125329 29566419

[20] Maaser C Sturm A Vavricka S , et al. ECCO-ESGAR guideline for diagnostic assessment in IBD Part 1: initial diagnosis, monitoring of known IBD, detection of complications. J Crohn's Colitis 2018; 13: 144–164. 10.1093/ecco-jcc/jjy113 30137275

[21] Rcr.ac.uk. 2021, https://www.rcr.ac.uk/system/files/publication/field_publication_files/BFCR(14)17_Standards_ultrasound.pdf (accessed 24 January 2022).

[22] Simcock IC Reeve R Burnett C , et al. Clinical academic radiographers: a challenging but rewarding career. Radiography 2021; 27: 14–19. 34274226 10.1016/j.radi.2021.06.008

